# Case Report: Severe Community-Acquired Pneumonia in Réunion Island due to *Acinetobacter baumannii*

**DOI:** 10.4269/ajtmh.23-0820

**Published:** 2024-06-04

**Authors:** Giacomo Rotini, Axel de Mangou, Agathe Combe, Julien Jabot, Bérénice Puech, Laurence Dangers, Mathilde Nativel, Nicolas Allou, Guillaume Miltgen, Charles Vidal

**Affiliations:** ^1^ Department of Intensive Care Medicine, Felix Guyon University Hospital, Saint-Denis, France;; ^2^ Department of Microbiology, Felix Guyon University Hospital, Saint-Denis, France;; ^3^ UMR PIMIT, CNRS 9192, INSERM U1187, IRD 249, University of Reunion, Saint-Clotilde, France

## Abstract

*Acinetobacter baumannii* (*Ab*) is a well-known nosocomial pathogen that has emerged as a cause of community-acquired pneumonia (CAP) in tropical regions. Few global epidemiological studies of CAP*-Ab* have been published to date, and no data are available on this disease in France. We conducted a retrospective chart review of severe cases of CAP-*Ab* admitted to intensive care units in Réunion University Hospital between October 2014 and October 2022. Eight severe CAP*-*Ab cases were reviewed. Median patient age was 56.5 years. Sex ratio (male-to-female) was 3:1. Six cases (75.0%) occurred during the rainy season. Chronic alcohol use and smoking were found in 75.0% and 87.5% of cases, respectively. All patients presented in septic shock and with severe acute respiratory distress syndrome. Seven patients (87.5%) presented in cardiogenic shock, and renal replacement therapy was required for six patients (75.0%). Five cases (62.5%) presented with bacteremic pneumonia. The mortality rate was 62.5%. The median time from hospital admission to death was 3 days. All patients received inappropriate initial antibiotic therapy*. Acinetobacter baumannii* isolates were all susceptible to ceftazidime, cefepime, piperacillin-tazobactam, ciprofloxacin, gentamicin, and imipenem. Six isolates (75%) were also susceptible to ticarcillin, piperacillin, and cotrimoxazole. Severe CAP-*Ab* has a fulminant course and high mortality. A typical case is a middle-aged man with smoking and chronic alcohol use living in a tropical region and developing severe CAP during the rainy season. This clinical presentation should prompt administration of antibiotic therapy targeting *Ab*.

## INTRODUCTION

*Acinetobacter baumannii* (*Ab*) is a gram-negative, nonfermenting coccobacillus typically found in water and soil. This nosocomial pathogen^1^ is known to cause ventilator-associated pneumonia and is associated with prolonged length of hospital stay and high mortality.^2^^,^^3^ Over recent decades, *Ab* has also emerged as a rare cause of community-acquired pneumonia (CAP) in tropical and subtropical regions. Community-acquired pneumonia due to *Ab* (CAP-*Ab*) often has a fulminant course, with or without bloodstream infection,^4^ and is associated with very high mortality rates.^5^ The following risk factors for severe CAP-*Ab* have been identified: chronic alcohol use, smoking, chronic lung disease, chronic renal disease,^6^ and diabetes mellitus.^4^ Few global epidemiological studies on CAP-*Ab* have been published to date,^5^^–^^10^ and no data are available on this disease in France. The aim of this case series report was to describe the clinical and microbiological characteristics of patients with severe CAP-*Ab* in Réunion Island, a French overseas department located in the tropical Indian Ocean region.

## MATERIALS AND METHODS

### Study sample.

All patients with a confirmed diagnosis of severe CAP-*Ab* admitted to the two intensive care units (ICUs) of Réunion Island University Hospital (Saint-Denis and Saint-Pierre sites) between October 2014 and October 2022 were retrospectively evaluated. The following definitions were applied: Community-acquired pneumonia was defined as pneumonia acquired outside the hospital and diagnosed within 48 hours of hospital admission, in accordance with French guidelines.^11^^,^^12^ A diagnosis of severe CAP was established according to the guidelines of the American Thoracic Society.^13^ The rainy season was defined as the period from November to April.^14^ Chronic alcohol use was defined by an Alcohol Use Disorders Identification Test (AUDIT-C) score greater than or equal to 10.

### Microbiological investigations.

Blood cultures and respiratory samples (sputum samples from non-intubated patients and tracheobronchial aspirates, bronchoalveolar lavages, or protected distal samples from intubated patients) were systematically investigated. Microorganism identification was performed by Gram staining and definite identification by culturing. Identification was carried out using matrix-assisted laser desorption ionization time-of-flight mass spectrometry. Antimicrobial susceptibility testing was assessed by the disk diffusion method or minimum inhibitory concentration determination using European Committee on Antimicrobial Susceptibility Testing breakpoints.

## STATISTICAL ANALYSES

Data were expressed as total number (percentage) for categorical variables and as median (interquartile range [IQR]) for continuous variables.

### Ethics and approval.

This observational study was approved by the French Ethics Committee of Infectious Diseases and Tropical Medicine and was declared to the French National Commission for Data Protection and Liberties (French Data Protection Agency; #2226468). A written notice describing the data collection process was provided to all participants or their legally authorized representative.

## RESULTS

Eight cases of severe CAP-*Ab* were included during the 8-year study period. Case summaries are shown in the Supplemental Materials. Patients with a hospital-acquired *Ab* infection were excluded (*N* = 50).

### Baseline characteristics.

In our eight CAP-*Ab* cases, six patients were male (75%) and two were female (25%). The median age was 56.5 years (IQR: 50–59 years). Almost all patients (seven of eight, 87.5%) were active smokers. Six patients (75%) had chronic alcoholism (AUDIT-C score ≥10). None of our patients had diabetes mellitus. Some patients had other underlying diseases such as chronic obstructive pulmonary disease, high blood pressure, cirrhosis, or immunosuppression. One had active cancer. Regarding jobs and potential exposure, there was one farmer (living in substandard housing), one tiler, one cleaning lady, one unemployed patient, one retired mechanic, one retired policeman, one trader working in Madagascar (last trip there was 2 years previously), and one welder. Patient characteristics are shown in Table 1.

**Table 1:** Demographics, clinical features, and outcomes of eight episodes of community-onset *Acinetobacter baumannii* pneumonia in Réunion Island (2014–2022)

Parameters CAP-*Ab*	Value (*N* = 8)
Demographics	
Age (years)	56.5 [50–59]
Male Sex	6 (75)
BMI (kg/m_2_)	21.5 [18–22]
Rainy Season	6 (75)
Comorbidities	
Diabetes Mellitus	0
Chronic Alcohol Use	6 (75)
Tobacco Use	7 (87.5)
Chronic Lung Disease	3 (37.5)
HBP	2 (25)
Chronic Renal Failure	0
Chronic Liver Disease	1 (12.5)
Immunosuppression	1 (12.5)
Symptoms in Previous 48 Hours	
Cough	3 (37.5)
Dyspnea	5 (62.5)
Fever	6 (75)
Pleuritic Chest Pain	3 (37.5)
Productive Cough	2 (25)
Blood-Stained Sputum	1 (12.5)
Cachexia	5 (62.5)
Time from Symptom Onset to Presentation, days	5 [2.5–10.5]
Severity	
SAPS II	63 [52.5–77.5]
SOFA Score	9 [7–10]
CURB-65	2 [1–3]
Septic Shock	8 (100)
Cardiogenic Shock	7 (87.5)
ARDS	8 (100)
Severe ARDS	6 (75)
PaO_2_/FiO_2_ (nadir), mm Hg	80 [75.5–105]
RRT	6 (75)
Outcomes	
ICU Length of Stay, days	2.5 [2–11]
Length of MV, days	2.5 [2–7]
Mortality	5 (62.5)
Time to Death after Hospitalization, days	3 [2–4]

ARDS = acute respiratory distress syndrome; BMI = body mass index; CAP-*Ab* = community-acquired pneumonia due to *Acinetobacter baumannii*; HBP = high blood pressure; ICU = intensive care unit; MV = mechanical ventilation; PaO_2_/FiO_2_ = ratio of arterial oxygen partial pressure to fractional inspired oxygen; RRT = renal replacement therapy; SAPS II = Simplified Acute Physiology Score II; SOFA = Sequential Organ Failure Assessment. Data are expressed as number (%) or median [interquartile range]. Chronic lung disease is defined as a recorded diagnosis of chronic obstructive pulmonary disease or bronchiectasis. Chronic alcohol use is defined by an AUDIT-C score ≥10. Chronic liver disease is defined as a recorded diagnosis of cirrhosis and chronic renal failure by the need for hemodialysis. Severe ARDS is defined by a PaO_2_/FIO_2_ ratio ≤100 mm Hg. CURB-65 score for pneumonia severity Alcohol Use Disorders Identification Test (AUDIT-C) score Ratio of arterial oxygen partial pressure to fractional inspired oxygen below 100 mm Hg (PaO_2_/FiO_2_).

### Clinical presentation and outcomes.

Six cases (75%) occurred during the rainy season (November to April) and five cases during the rainiest months of the year (January to April). Most CAP-*Ab* patients presented with acute onset of fever, shortness of breath, and cachexia (including asthenia, anorexia, and/or weight loss). Few patients (two of eight, 25%) had sputum production. At admission, CAP-*Ab* patients had a propensity to low absolute leukocyte count, low platelet count, and elevated lactate levels. Laboratory findings on ICU admission are summarized in Table 2. All patients progressed to septic shock and acute respiratory distress syndrome (ARDS). Six patients (75%) presented with severe ARDS, defined as ratio of arterial oxygen partial pressure to fractional inspired oxygen below 100 mm Hg (PaO_2_/FiO_2_). Seven patients (87.5%) presented with cardiogenic shock assessed by bedside cardiac ultrasonography and required the use of dobutamine. Six patients (75%) needed renal replacement therapy (RRT) for severe acidosis. The median Sequential Organ Failure Assessment score was 9, and the median Simplified Acute Physiology Score II was 63 (Table 1). The mortality rate in our study was 62.5% (five of eight patients). The median time from hospital admission to death was 3 days (IQR: 2–4 days). Median ICU length of stay and duration of mechanical ventilation was 2.5 days (IQR: 2–11 and 2–7 days, respectively).

**Table 2:** Laboratory findings

Parameters	CAP-*Ab* (*N* = 8)
Laboratory Findings on ICU Admission	
Hemoglobin, g/dL	13 [10.35–14]
Absolute Leukocyte Count, G/L	2.15 [0.57–3.84]
Platelet Count, G/L	103 [82.5–144]
Creatinine Levels, *µ*mol/L	93 [73.5–129.5]
Bilirubin Levels, *µ*mol/L	23.5 [9–40.5]
Prothrombin Time, %	65 [46–73]
Lactate levels, mmol/L	4.4 [3.05–6.2]
Creatine Phosphokinase Levels, mg/dL	294 [181–1,126]
Alanine Aminotransferase, UI/L	36 [31.5–60.5]
Troponin Levels, ng/mL	23 [17–370]
C-Reactive Protein Levels, mg/L	135 [74.5–325.5]
Bacteremia	5 (62.5)

CAP-*Ab* = community-acquired pneumonia due to *Acinetobacter baumannii*; ICU = intensive care unit. Data are expressed as number (%) or median [interquartile range].

### Treatment and antimicrobial susceptibility.

Most patients (five of eight, 62.5%) had *Ab* bacteremia on presentation. Five Gram stains (blood culture and/or respiratory samples) showed gram-negative bacilli. One showed gram-positive cocci and gram-negative bacilli in sputum. Two were noncontributory. All cultures became positive to *Ab*. Regarding respiratory samples, three patients underwent tracheobronchial aspiration, two bronchoalveolar lavage, and three protected specimen brushing. No other pathogenic species were cultured in the samples. The *Ab* isolates were all susceptible to ceftazidime, cefepime, piperacillin-tazobactam, ciprofloxacin, gentamicin, and imipenem. Many (six of eight, 75%) were also susceptible to ticarcillin, piperacillin, and cotrimoxazole. Strain susceptibilities are shown in Table 3. All patients received inappropriate initial antibiotic therapy. Six patients (75%) received a third-generation cephalosporin (ceftriaxone or cefotaxime) and a macrolide. One received ceftriaxone alone, and one received amoxicillin-clavulanic acid. Treatment was adapted considering clinical severity and/or germ identification. The mean delay between hospital admission and effective antibiotic therapy was greater than 12 hours.

**Table 3:** Antimicrobial susceptibility testing of the eight isolates causing *Acinetobacter baumannii* community-acquired pneumonia

Antibiotics	Susceptible	Intermediate	Resistant
Ticarcillin	6 (75)	2 (25)	0
Piperacillin	6 (75)	1 (12.5)	1 (12.5)
Piperacillin-Tazobactam	8 (100)	0	0
Ceftazidime*	7 (100)	0	0
Cefepime	8 (100)	0	0
Imipenem	8 (100)	0	0
Gentamicin	8 (100)	0	0
Co-trimoxazole	6 (75)	1 (12.5)	1 (12.5)
Ciprofloxacin	8 (100)	0	0

Data are expressed as number (%).

* *N* = 7 owing to lack of data.

A summary is shown in Supplemental Figure 1.

## DISCUSSION

This work is the first to describe the clinical and microbiological characteristics of patients with severe CAP-*Ab* in Réunion Island, a French overseas department located in the tropical Indian Ocean. Our case series complements the literature on CAP-*Ab* in tropical and subtropical regions.

The eight cases of severe CAP-*Ab* described in this work occurred over a period of 8 years, which corresponds to an incidence of approximately 0.1 case per 100,000 people/year. This figure is lower than that reported in other tropical regions. Thus, in tropical parts of Australia^6^^,^^10^ and East Asia,^5^^,^^8^^,^^9^ the incidence has ranged from 0.6 to 1.8 cases per 100,000 people/year.^5^^,^^6^^,^^10^ Consistent with other studies,^4^^,^^5^ the main clinical signs of severe CAP-*Ab* in our series were fever, dyspnea, cough, and chest pain. Most of our patients (75.0%) developed severe CAP-*Ab* during the rainy season, including one man who was transferred from the neighboring island of Mayotte (case #1) where the rainy season lasts from December to April. A warm moist climate has been shown to be a factor of CAP-*Ab*.^4^^,^^7^^–^^9^^,^^15^^–^^17^ In a major prospective study conducted in tropical Australia, 83.0% of patients hospitalized for CAP-*Ab* had contracted the disease during the rainy season.^6^

Mortality in our series was very high at 62.5%, a figure considerably higher than that reported for severe CAP caused by other microorganisms.^18^^–^^21^ Other studies of CAP-*Ab* have reported mortality rates ranging from 42.0% to 64.0%, with a median of 58.0%.^5^^,^^7^^–^^10^^,^^15^^,^^22^ The very high mortality rate observed in our series is likely related to patient severity on admission (median Simplified Acute Physiology Score II of 63; median Sequential Organ Failure Assessment score of 9; the CURB-65 [confusion, uremia, respiratory rate, BP, age ≥ 65 years] score of 2). The median time from hospital admission to death was 3 days, confirming that the disease may have a fulminant course.^5^

All patients in our series progressed to septic shock.^23^ One study of CAP-*Ab* found a similar rate of septic shock (92.0%),^8^ whereas others reported lower rates (from 58.0% to 75.0%).^5^^,^^6^ The frequency of RRT has been shown to rise with the frequency of septic shock.^15^ In our series, 75.0% of cases required RRT for severe metabolic acidosis.^24^ By contrast, in the study by Leung et al.,^5^ 15.8% of patients with CAP-*Ab* required RRT for acute renal failure. Other studies have reported rates of acute renal failure ranging from 49.0% to 75.0%, though without specifying whether evaluated patients received RRT.^6^^,^^9^ The discrepancy in reported rates of RRT in patients with CAP-*Ab* may be explained by the fact that policies for the management of acute renal failure or septic shock vary greatly across ICUs.^25^^,^^26^ Cardiogenic shock^27^ occurred in 87.5% of our patients, all of whom had suspected acute septic myocarditis. To our knowledge, only one study has described cardiogenic shock in patients with CAP-*Ab*.^15^ The increasing use of bedside ultrasound examination could, in the future, result in more frequent observations of cardiogenic shock in patients with severe CAP-*Ab*. All patients in our series rapidly progressed to ARDS.^28^ Similarly, other studies of CAP-*Ab* have reported rates of ARDS ranging from 75.0% to 88.0%.^5^^,^^6^^,^^9^^,^^10^

Consistent with other studies,^5^^,^^6^ five of our patients (62.5%) developed bacteremic pneumonia. This is an interesting finding, as bacteremia has been shown to be a poor prognostic factor in patients with CAP.^29^^,^^30^ Three patients (37.5%) in our series had disseminated intravascular coagulation,^31^ though it should be noted that data on D-dimer levels were missing for the other five patients. Rates of disseminated intravascular coagulation ranging from 58.0% to 75.0% have been reported in patients with CAP-*Ab*.^5^^,^^9^ As in other studies, thrombocytopenia and leukopenia were very common in our patients.^5^^,^^7^^,^^22^

Half of the patients in our series were middle-aged men, which is consistent with other studies.^6^^,^^7^ Moreover, 75.0% were chronic alcohol users and 87.5% were smokers; one patient had no history of smoking or chronic alcohol use, and another was a former alcoholic. Alcohol use has also been a risk factor for CAP-*Ab* in Australia and to a lesser extent in East Asia.^2^^,^^4^^,^^17^ Studies have described how alcohol consumption reduces immunity and/or increases *Ab* carriage in the throat of patients.^16^^,^^32^^,^^33^ Leung et al.^5^ reported a statistically higher rate of smoking in patients with CAP-*Ab* compared with patients with hospital-acquired *Ab* pneumonia.^5^ In their review of case series of CAP-*Ab*, Dexter et al.^4^ found considerable variation in the frequency of smoking. In contrast to other studies,^6^^,^^8^^–^^10^ none of the patients in our series had diabetes mellitus. This finding is especially surprising given that the prevalence of diabetes mellitus is much higher in Réunion Island than in mainland France. It could be explained by underdiagnosis, as our patients were less likely to consult health services because of their frequent alcohol use. Another possible explanation is that diabetes mellitus does not constitute a risk factor for CAP-*Ab* in Réunion Island. Indeed, as the review by Dexter et al.^4^ indicated, risk factors for CAP-*Ab* can vary across regions.

Community-acquired pneumonia is typically caused by pathogens found in temperate regions, including *Streptococcus pneumoniae*, *Legionella* spp., and the *influenza* virus.^34^ To date, the etiology of CAP in Réunion Island has been investigated in only one comprehensive study.^10^ As a result, severe CAP is currently managed based on French and international guidelines,^12^^,^^13^ which do not specifically target microorganisms found in tropical regions (e.g., *Burkholderia pseudomallei* and^35^^–^^37^ *Yersinia pestis*^38^). These guidelines recommend initiating antibiotic therapy effective against all strains of *S. pneumoniae* and *Legionella* spp., namely combination therapy with cephalosporin and a macrolide or monotherapy with a respiratory fluoroquinolone. *Acinetobacter baumannii* strains are, however, intrinsically resistant to macrolides and third-generation cephalosporins (with the notable exception of ceftazidime).^39^ When CAP-*Ab* is suspected, we suggest using cefotaxime with levofloxacin. Better knowledge of the clinical and microbiological characteristics of CAP-*Ab* in Réunion Island and other tropical regions could improve the management of affected patients while accounting for seasonality.

Five wild strains of *Ab* were identified in our series. The eight isolated strains were susceptible to ceftazidime, cefepime, piperacillin-tazobactam, and ciprofloxacin (Table 3), a finding consistent with the literature.^6^^,^^9^^,^^15^^,^^40^^,^^41^ All patients in our series received inappropriate initial antibiotic therapy. The importance of early appropriate treatment to improve the outcome of bacterial infections is well-known.^6^^,^^23^^,^^42^^,^^43^ Iwasawa et al.^44^ have suggested using Gram staining for the early identification of *Ab*. In our series, five Gram stain tests suggested that *Ab* was a causative pathogen of CAP, showing gram-negative bacilli. More effective methods for the identification of *Ab* include multiplex real-time polymerase chain reaction^45^^,^^46^ and matrix-assisted laser desorption ionization time-of-flight mass spectrometry.^47^ Next-generation sequencing^48^ could also be used for identification, though cost, lead time, and availability may constitute obstacles to the adoption of this technique.

Ultimately, improving patient outcome will require expert advice from infectious diseases specialists and pharmacologists.^47^^,^^49^^,^^50^ Individual protocols for at-risk patients who develop severe pneumonia during the rainy season may also be needed.^6^ Future studies should compare severe CAP caused by *Ab* versus other pathogens to help determine the risk factors for severe CAP-*Ab* in Réunion Island.

## CONCLUSION

Severe CAP-*Ab* often has a fulminant course and is associated with very high mortality. A typical case is that of a middle-aged man with a history of smoking and chronic alcohol use who lives in a tropical region and develops severe pneumonia during the rainy season. This clinical presentation should prompt administration of antibiotic therapy targeting *Ab*.

## Supplemental Materials

10.4269/ajtmh.23-0820 Supplemental Materials
