# Supplementary material for: Case Report: Severe Community-Acquired Pneumonia in Réunion Island due to Acinetobacter baumannii
Source: Am J Trop Med Hyg. 2024 Jun 4;111(1):136–40. doi: 10.4269/ajtmh.23-0820 (PMC11229653; doi:10.4269/ajtmh.23-0820)
Supplement: Supplemental Materials [file tpmd230820.SD1.pdf]

Supporting information

Case summaries

Case #1: In February 2016, a 21-year-old man with no medical history was admitted to Mayotte Hospital Center after 2 weeks of cachexia. The patient presented with typical pneumonia with fever, dyspnea, and tachycardia. He had no history of smoking or chronic alcohol use. He worked as a tiler. Clinical examination showed purpura with hematuria, hemorrhagic blebs on the cheeks, and pharyngitis. Laboratory results showed hyperleukocytosis (absolute leukocyte count 123.0 G/L), thrombocytopenia (platelet count 38 G/L), anemia (hemoglobin levels 6.5 g/dL), hyperlactatemia (lactate levels 3.5 mmol/L), rhabdomyolysis (creatine phosphokinase levels 1,126 UI/L), and hepatitis (aspartate transaminase (AST) /alanine transaminase (ALT) 134/72 UI/L). A chest radiograph revealed patchy consolidation in the right lower lobe and the left lung. Antibiotic therapy with ceftriaxone was initiated and changed to piperacillin-tazobactam and gentamicin a few hours later. On day 2, the patient was transferred to the hematology department of Saint-Denis University Hospital in Reunion Island for suspicion of acute promyelocytic leukemia (APL). His circulatory and respiratory status deteriorated, which led to his transfer to ICU on day 3. He was initially treated with non-invasive ventilation and rapidly required intubation with high ventilatory support and catecholamines. His status deteriorated further, with presence of a severe acute respiratory distress syndrome (ARDS), prompting the administration of neuromuscular blocking agents, prone positioning, and inhaled nitric oxide. The patient also required higher doses of noradrenaline (norepinephrine up to 8 µg/kg/min) and the addition of dobutamine. The presence of severe metabolic acidosis led to use of renal replacement therapy (RRT). No germs were found in direct examination of the bronchoalveolar lavage. A whole-body computed tomography (CT) scan showed pleural and pericardial effusion, lower lobe pneumonia, as well as multiple pulmonary, renal, hepatic, and splenic embolisms. Right thoracentesis removed 550 mL of sterile pleural fluid (with abnormal cells) and 220 mL of sterile pericardial fluid. Histological analyses confirmed the diagnosis of APL. Chemotherapy with cytarabine and idarubicin was initiated, along with preventive therapy (rasburicase). After identification of *A. baumannii* and *Candida glabrata* in respiratory cultures, antibiotic therapy was changed to meropenem, colomycin, and caspofungin on day 3. The results of antimicrobial susceptibility testing are shown in Appendix 1. Despite optimal management, the evolution was unfavorable, with multiple organ failure leading to death on day 5.

Case #2: In April 2016, a 49-year-old woman presented to the emergency department of Saint-Denis University Hospital after 7 days of progressive dyspnea, fever, and cough and 1 day of left chest pain. The patient had stopped drinking alcohol 11 years earlier. She had a history of smoking (30 pack-year) and peptic esophagitis. In 2011, she had developed CAP, which had been successfully treated with amoxicillin-clavulanic acid. She worked as a cleaning lady. Oxygen therapy (15 L/min) was initiated on admission. Laboratory results showed elevated procalcitonin levels (5.47 µg/L) and D-dimer levels (1,780 µg/L). A chest CT scan revealed left superior lobe pneumoniae and diffuse emphysema, with no evidence of pulmonary embolism. The patient was started on antibiotic therapy with ceftriaxone and spiramycin and then transferred to ICU. High-flow nasal cannula (HFNC) oxygen therapy was initiated, and antibiotic therapy was changed to piperacillin-tazobactam. Arterial blood gas analysis showed respiratory acidosis (pH 7.12; pCO_2 85 mmHg). The patient was intubated and required RRT. No germs were found in the direct examination of the protected distal sample. Despite protective mechanical ventilation and several prone positioning sessions, severe ARDS persisted, and higher doses of noradrenaline were needed. The patient presented with left ventricular dysfunction (left ventricular ejection fraction (LVEF)<20%), which prompted the administration of adrenalin up to 3 µg/kg/min. On day 2, *A. baumannii* was identified on respiratory samples and blood cultures collected on admission (Appendix 2). Venoarterial extracorporeal membrane oxygenation was started on day 2, but did not prevent multiple organ failure. Death occurred on day 3.

Case #3: In December 2017, a 59-year-old woman with a body mass index (BMI) of 14 kg/m^2 was admitted to the emergency department of a peripheral hospital in Reunion Island for alcohol withdrawal delirium with seizures. The patient had a history of chronic alcohol use, high blood pressure, and smoking (40 pack-year). She was unemployed. She presented with confusion and tachycardia (130 bpm). Blood alcohol level was 0.55 g/L. Diazepam and vitamin therapies were initiated. On the night of day 1, the patient developed acute respiratory failure associated with right chest pain and hypotension. She received a high concentration oxygen mask and 2 liters of isotonic fluid. A chest CT scan showed right lower lobe pneumonia. After her circulatory and respiratory status deteriorated, the patient was intubated and received noradrenaline up to 5 mg/h. She was started on ceftriaxone and spiramycin and transferred to the ICU of Saint-Denis University Hospital. She presented with septic shock and oliguria on admission to ICU. Laboratory tests showed neutropenia (absolute neutrophil count 0.22 G/L), lymphopenia (lymphocyte count 0.25 G/L), thrombocytopenia (platelet count 78 G/L), and elevated AST levels (205 UI/L). Arterial blood gas analysis found metabolic acidosis (pH 6.99) with hyperlactatemia (lactate levels 5.2 mmol/L). Gram staining of the tracheobronchial aspirate revealed numerous Gram-negative bacilli and Gram-positive cocci with some yeasts. On day 2, the patient was started on piperacillin-tazobactam and gentamicin. Renal replacement therapy was initiated, and bicarbonate fluids were used. The evolution was rapidly unfavorable. The patient progressed to refractory shock despite receiving noradrenaline (up to 15 µg/kg/min), dobutamine (up to 5 µg/kg/min), and fluids (ringer's lactate and albumin solution). The use of protective ventilation and cisatracurium in front of severe ARDS, did not prevent the worsening of hypoxemia. Death occurred on day 3. The tracheobronchial aspirate was positive for *A. baumannii* postmortem (Appendix 3).

Case #4: In April 2018, a 51-year-old man was admitted to the emergency department of Saint-Pierre University Hospital for fever and dyspnea that had been ongoing two days. The patient had a history of smoking (20 pack-year), chronic alcohol use, and cirrhosis (Child B) due to chronic hepatitis C. In 2010, he had developed a pulmonary abscess caused by *Escherichia coli* and complicated by pleural empyema. He worked as a trader. On admission, he presented with dyspnea requiring oxygen therapy (4 L/min). Laboratory tests showed acute renal failure (creatinine levels 219 µmol/L); elevated AST levels (80 UI/L), and leukopenia (absolute leukocyte count 3 G/L). Arterial blood gas analysis found hypoxemia (Pa02 60 mmHg) and hyperlactatemia (lactate levels 2.6 mmol/L). A chest radiograph showed infiltration of the left lung (Appendix 4b). The patient was started on cefotaxime and spiramycin and then transferred to ICU. The clinical examination found a respiratory rate of 30 breaths/minute with signs of struggle and coarse inspiratory crackles in the left lung. On day 2, the patient was intubated for respiratory failure du to ARDS and switched to piperacillin-tazobactam. Respiratory and blood samples collected on admission were positive for *A. baumannii* (Appendix 4a). Antibiotic therapy was changed to piperacillin alone for 10 days. The patient received RRT until renal recovery (weaning on day 12). He was extubated on day 8. Colitis due to *Clostridium difficile* was diagnosed and treated with metronidazole. The patient was transferred to the nephrology department on day 14 and was discharged from hospital on day 20.

Case #5: In January 2019, a 66-year-old man was admitted to the emergency department of a peripheral hospital in Reunion Island with productive cough and fever that had been ongoing for 3 days. The patient had a history of smoking, chronic alcohol use, and acute alcoholic hepatitis. The patient had developed ENT neoplasia in 2014. He was a retired mechanic. On admission, he presented with fever, tachycardia, and dyspnea. A chest radiograph showed right lower lobe pneumonia. Antibiotic therapy with amoxicillin-clavulanic acid was initiated. The patient received 1.5 liters of crystalloid fluids for hypotension and a high concentration oxygen mask followed by non-invasive ventilation. On the night of day 1, he received invasive mechanical ventilation and noradrenaline and was transferred to the ICU of Saint-Denis University Hospital. Laboratory results showed leukopenia (absolute leukocyte count 0.6 G/L), thrombocytopenia (platelet count 88.0 G/L), acute renal failure (creatinine levels 98.0 µmol/L; blood urea nitrogen levels 10.3 mmol/L), elevated AST levels (215 UI/L), respiratory acidosis (pH 7.17; pCO2 50 mmHg), and hyperlactatemia (lactate

levels 6.3 mmol/L). The patient presented with septic shock and tachycardia due to persistent atrial fibrillation. A chest radiograph showed a white right lung and a chest CT scan revealed lower lobe pneumonia, with predominance of the right lobe (Appendix 5b). Gram-positive bacilli and Gram-positive cocci were detected on bronchoalveolar lavage. On day 2, the patient was switched to cefotaxime, spiramycin, metronidazole, and amikacin. He presented with cardiac arrest (no-flow 0 min; low-flow 3 min) and was resuscitated with adrenalin (1 mg). Over the next 12 hours, he received noradrenaline (3 µg/kg/min), dobutamine (15 µg/kg/min), as well as crystalloid and colloid fluids. Antibiotic therapy was changed to piperacillin-tazobactam. Metabolic acidosis with hyperlactatemia (lactate levels 10 mmol/L) persisted despite the initiation of RRT. The deteriorating respiratory status required recommended treatment for severe ARDS (protective ventilation, neuromuscular blocking agents, and prone positioning therapy). Respiratory samples were positive for *A. baumannii* (Appendix 5a). Death occurred on day 3 due to multiple organ failure.

Case #6: In March 2020, a 55-year-old man with a BMI of 19 kg/m^2 was admitted to the emergency department of a peripheral hospital in Reunion Island after 1 month of cachexia and 1 week of productive cough. The patient had a history of chronic alcohol use and smoking (40 pack-year). He was a retired policeman. Clinical examination showed dyspnea, tachycardia, and hypotension. A chest CT scan revealed left lower lobe pneumonia and diffuse emphysema. Laboratory results showed leukopenia (absolute leukocyte count 0.5 G/L), elevated AST levels (136 UI/L), and venous hyperlactatemia (11 mmol/L). High flow nasal cannula oxygen therapy (60 L/min, FiO2 60%) and fluid therapy were initiated. The patient was started on ceftriaxone and spiramycin. Arterial blood gas analysis found hypoxemia (Pa02 84 mmHg) and hyperlactatemia (lactate levels 6.3 mmol/L). The patient was transferred to the ICU of Saint-Denis University Hospital for suspected SARS-CoV2 infection, where he was intubated and mechanically ventilated. He rapidly developed severe ARDS. Amikacin and cefotaxime were added to the antibiotic regimen. The patient developed left ventricular dysfunction (LVEF<15%), which required higher doses of catecholamines and the addition of dobutamine. Renal replacement therapy was initiated. Gram staining revealed numerous Gram-negative bacilli. Antibiotic therapy was changed to piperacillin-tazobactam and amikacin. No evidence of SARS-CoV2 infection was found. The patient rapidly progressed to multiple organ failure despite optimal management. Death occurred on day 2. The tracheobronchial aspirate was positive for *A. baumannii* postmortem (Appendix 6).

Case #7: In May 2022, a 58-year-old man called emergency services for dyspnea and chest pain. The patient had fallen from a height the previous day, following approximately 3 weeks of productive cough and cachexia. He had a history of chronic alcohol use and smoking (50 pack-year). He work as farmer. On admission to the emergency department of Saint-Denis University Hospital, he presented with fever, respiratory failure, and hypotension requiring fluid therapy. A chest radiograph showed left lung infiltration. Antibiotic therapy with ceftriaxone and spiramycin was initiated. Dyspnea and hypoxemia persisted despite the use of a high concentration oxygen mask. The patient was transferred to ICU, where he was rapidly intubated. Laboratory results showed thrombocytopenia (platelet count 118 G/L) and elevated D-dimer levels (15,142 µg/L). The patient received the recommended treatment for severe ARDS. He then presented with septic shock, which prompted the administration of catecholamines up to 10 µg/kg/min. Direct exam showed some Gram-positive bacilli and numerous Gram-negative bacilli on pulmonary sample; and Gram-negative bacilli on blood sample. Multi-drug sensitive *A. baumannii* was detected in blood and respiratory samples (Appendix 7). After 24 hours of inappropriate antibiotic therapy, the patient was switched to ceftazidime (7 days) and amikacin (2 days). On day 2, he presented with cardiogenic shock likely due to septic myocarditis, which prompted the administration of dobutamine. On day 4, catecholamines were stopped and diuretic therapy was initiated (for 4 days). On day 7, the patient was successfully weaned from mechanical ventilation. The fever stopped and the disseminated intravascular coagulation observed on admission to ICU began to regress. The patient was discharged from ICU on day 10.

Case #8: In October 2022, a 59-year-old man called emergency services after 3 days of cachexia and 1 day of dyspnea and chest pain. He had a history of chronic alcohol use, smoking, and limping after a

fall from a ladder. He worked as a welder. On admission to the emergency department of Saint-Denis University Hospital, he presented with fever and hypotension requiring fluid therapy. He developed respiratory failure, which persisted despite the use of a high concentration oxygen mask and HFNC oxygen therapy. In view of this, he was intubated and transferred to ICU. Antibiotic therapy with cefotaxime and spiramycin was initiated. A chest CT scan revealed multilobar pneumonia with infiltration of the entire right lung and the left lower lobe, diffuse emphysema, and bronchiectasis. Laboratory results showed leukopenia (absolute leukocyte count 1.3 G/L), hyperlactatemia (lactate levels 3.6 mmol/L), acute renal failure (creatinine levels 142 µmol/L; blood urea nitrogen levels 10.8 mmol/L), and elevated D-dimer levels (3,162 µg/L). The patient received the recommended treatment for severe ARDS. He presented with septic shock requiring the use of catecholamines up to 3 µg/kg/min and left ventricular dysfunction (LVEF=30%) prompting the administration of dobutamine up to 5 µg/kg/min. Direct exam showed some Gram-positive bacilli and numerous Gram-negative bacilli on pulmonary sample; and Gram-negative bacilli on blood sample. On day 2, multi-drug sensitive *A. baumannii* was detected in blood and respiratory samples (Appendix 8). After 24 hours of inappropriate antibiotic therapy, the patient was switched to piperacillin (for 10 days). The patient received diuretic therapy for several days. Catecholamines were stopped on day 7. The biological inflammatory syndrome began to regress on day 11. The patient was successfully weaned from mechanical ventilation on day 13. He was discharged from ICU on day 16.

Appendix 1
Antimicrobial susceptibility testing of *Acinetobacter baumannii isolate* - Case #1

Penicillins	
Ticarcillin	Intermediate
Ticarcillin/clavulanic acid	Intermediate
Piperacillin	**Resistant**
Piperacillin/tazobactam	Susceptible
Cephalosporins (parenteral route)	
Ceftazidime	Susceptible
Cefepime	Susceptible
Monobactams	
Aztreonam	**Resistant**
Carbapenems	
Imipenem	Susceptible
Meropenem	Susceptible
Aminoglycosides	
Amikacin	Susceptible
Tobramycin	Susceptible
Gentamicin	Susceptible
Quinolones	
Ciprofloxacin	Susceptible
Sulfamides	
Co-trimoxazole (Trimethoprim/sulfamethoxazole)	**Resistant**
Other	
Fosfomycin	**Resistant**

Appendix 2
Antimicrobial susceptibility testing of *Acinetobacter baumannii isolate* - Case #2

Penicillins	
Ticarcillin	Susceptible
Ticarcillin/clavulanic acid	Susceptible
Piperacillin	Intermediate
Piperacillin/tazobactam	Susceptible
Cephalosporins (parenteral route)	
Ceftazidime	Susceptible
Cefepime	Susceptible
Monobactams	
Aztreonam	**Resistant**
Carbapenems	
Imipenem	Susceptible
Meropenem	Susceptible
Aminoglycosides	
Amikacin	Susceptible
Tobramycin	Susceptible
Gentamicin	Susceptible
Quinolones	
Ciprofloxacin	Susceptible
Sulfamides	
Co-trimoxazole (Trimethoprim/sulfamethoxazole)	Intermediate
Other	
Fosfomycin	**Resistant**

Appendix 3
Antimicrobial susceptibility testing of *Acinetobacter baumannii isolate* - Case #3

Penicillins	
Ticarcillin	Susceptible
Ticarcillin/clavulanic acid	Susceptible
Piperacillin	Susceptible
Piperacillin/tazobactam	Susceptible
Cephalosporins (parenteral route)	
Ceftazidime	Susceptible
Cefepime	Susceptible
Monobactams	
Aztreonam	**Resistant**
Carbapenems	
Imipenem	Susceptible
Meropenem	Susceptible
Aminoglycosides	
Amikacin	Susceptible
Tobramycin	Susceptible
Gentamicin	Susceptible
Quinolones	
Ciprofloxacin	Susceptible
Sulfamides	
Co-trimoxazole (Trimethoprim/sulfamethoxazole)	Susceptible
Other	
Fosfomycin	**Resistant**

Appendix 4a
Antimicrobial susceptibility testing of *Acinetobacter baumannii isolate* - Case #4

Penicillins	
Ticarcilline	Susceptible
Ticarcilline/ac. clavulanique	Susceptible
Piperacillin	Susceptible
Piperacillin/tazobactam	Susceptible
Cephalosporins (parenteral route)	
Ceftazidime	Susceptible
Cefepime	Susceptible
Carbapenems	
Imipenem	Susceptible
Meropenem	Susceptible
Aminoglycosides	
Amikacin	Susceptible
Tobramycin	Susceptible
Gentamicin	Susceptible
Quinolones	
Ciprofloxacin	Susceptible
Sulfamides	
Co-trimoxazole (Trimethoprim/sulfamethoxazole)	Susceptible

Appendix 4b

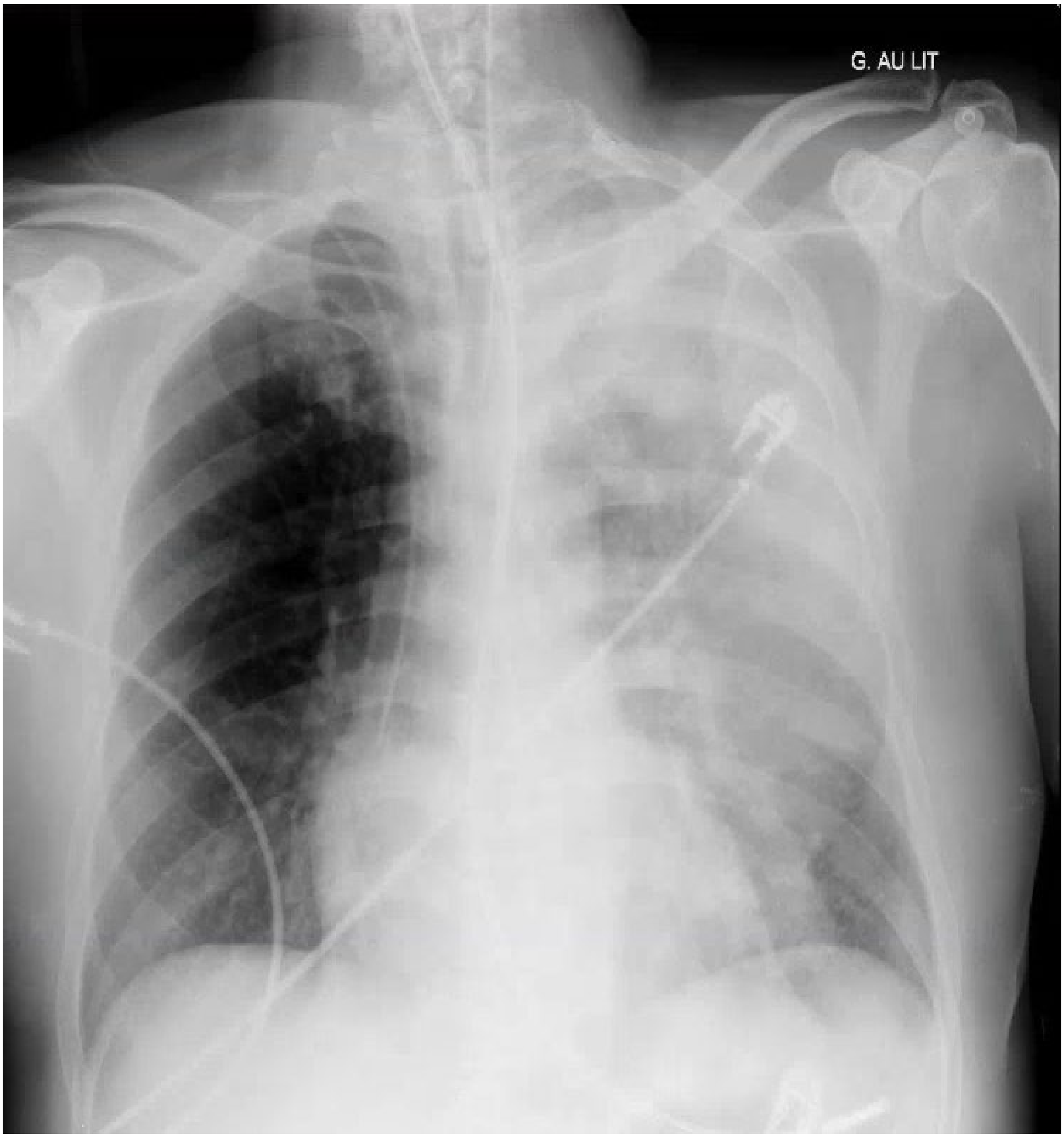

Appendix 5a
Antimicrobial susceptibility testing of *Acinetobacter baumannii isolate* - Case #5

Penicillins	
Ticarcillin	Intermediate
Ticarcillin/clavulanic acid	Intermediate
Piperacillin	Susceptible
Piperacillin/tazobactam	Susceptible
Cephalosporins (parenteral route)	
Cefepime	Susceptible
Monobactams	
Aztreonam	**Resistant**
Carbapenems	
Imipenem	Susceptible
Meropenem	Susceptible
Aminoglycosides	
Amikacin	Susceptible
Tobramycin	Susceptible
Gentamicin	Susceptible
Quinolones	
Ciprofloxacin	Susceptible
Sulfamides	
Co-trimoxazole (Trimethoprim/sulfamethoxazole)	Susceptible

Appendix 5b

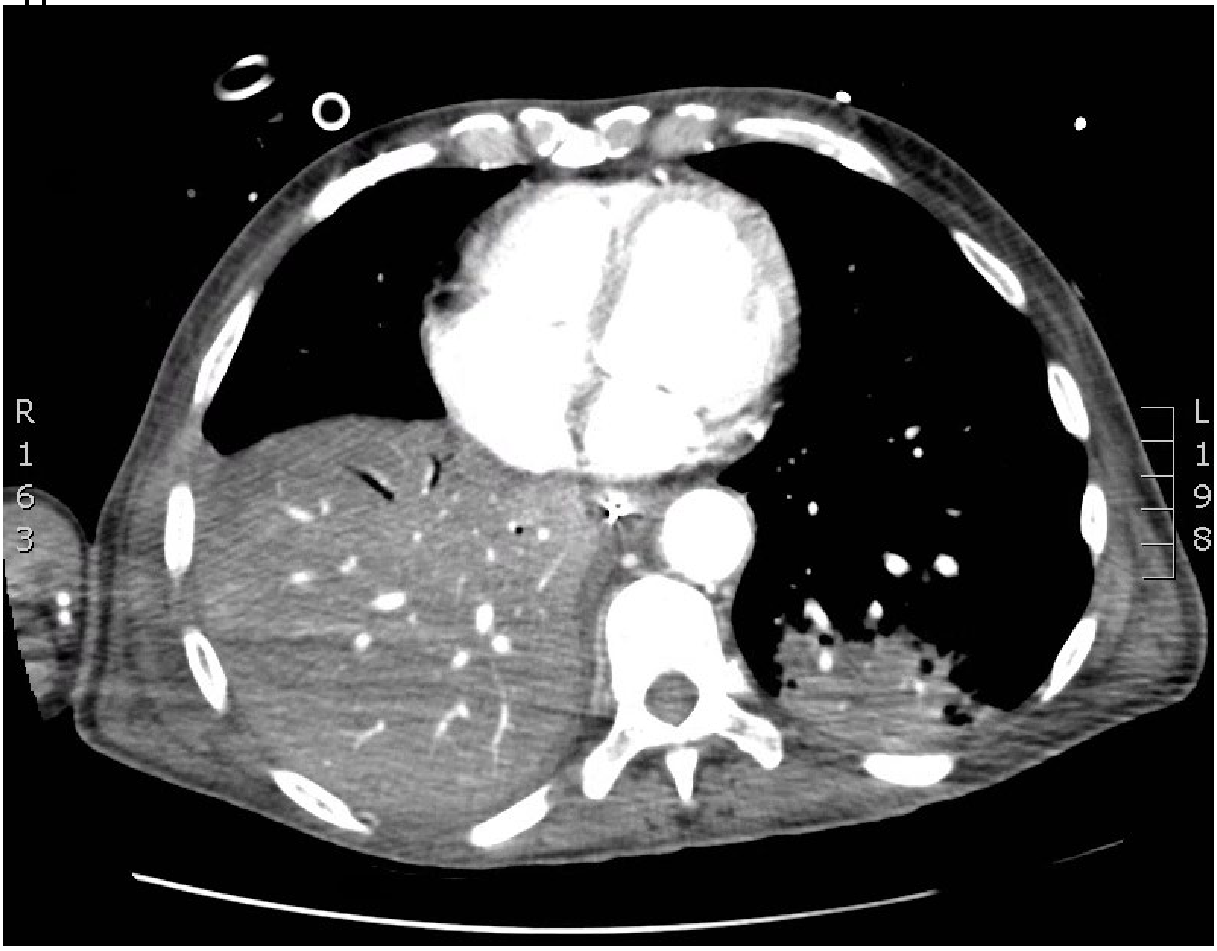

Appendix 6
Antimicrobial susceptibility testing of *Acinetobacter baumannii isolate* - Case #6

Penicillins	
Ticarcilline	Susceptible
Ticarcilline/ac. clavulanique	Susceptible
Piperacilline	Susceptible
Cephalosporins (parenteral route)	
Ceftazidime	Susceptible
Cefepime	Susceptible
Monobactams	
Aztreonam	**Resistant**
Carbapenems	
Imipenem	Susceptible
Meropenem	Susceptible
Aminoglycosides	
Amikacin	Susceptible
Tobramycin	Susceptible
Gentamicin	Susceptible
Quinolones	
Ciprofloxacin	Susceptible
Sulfamides	
Co-trimoxazole (Trimethoprim/sulfamethoxazole)	Susceptible

Appendix 7
Antimicrobial susceptibility testing of *Acinetobacter baumannii isolate* - Case #7

Penicillins	
Ticarcillin	Susceptible
Ticarcillin/clavulanic acid	Susceptible
Piperacillin	Susceptible
Piperacillin/tazobactam	Susceptible
Cephalosporins (parenteral route)	
Ceftazidime	Susceptible
Cefepime	Susceptible
Monobactams	
Aztreonam	**Resistant**
Carbapenems	
Imipenem	Susceptible
Meropenem	Susceptible
Aminoglycosides	
Amikacin	Susceptible
Tobramycin	Susceptible
Gentamicin	Susceptible
Quinolones	
Ciprofloxacin	Susceptible
Sulfamides	
Co-trimoxazole (Trimethoprim/sulfamethoxazole)	Susceptible

Appendix 8
Antimicrobial susceptibility testing of *Acinetobacter baumannii isolate* - Case #8

Penicillins	
Ticarcillin	Susceptible
Ticarcillin/clavulanic acid	Susceptible
Piperacillin	Susceptible
Piperacillin/tazobactam	Susceptible
Cephalosporins (parenteral route)	
Ceftazidime	Susceptible
Cefepime	Susceptible
Monobactams	
Aztreonam	**Resistant**
Carbapenems	
Imipenem	Susceptible
Meropenem	Susceptible
Aminoglycosides	
Amikacin	Susceptible
Tobramycin	Susceptible
Gentamicin	Susceptible
Quinolones	
Ciprofloxacin	Susceptible
Sulfamides	
Co-trimoxazole (Trimethoprim/sulfamethoxazole)	Susceptible

Figure S1. Synthesis.

Case	Year	Month	Area of origin	Age	Sex	Comorbidity	Presenting features	Delay of effective therapy (hours)	Initial antibiotic therapy	Effective antibiotic therapy	Death/ Time to death (days)
1	2016	February	Mayotte	21	Male	Immunosuppression	Septic shock, ARDS, RRT, cardiogenic shock	< 12	Ceftriaxone	Piperacillin-tazobactam and gentamicin	Yes / 5
2	2016	April	Reunion	49	Female	Tobacco use, COPD	Septic shock, ARDS, RRT, cardiogenic shock	> 12	Ceftriaxone and spiramycin	Piperacillin-tazobactam	Yes / 4
3	2017	December	Reunion	59	Female	Chronic alcoholism, tobacco use, HBP	Septic shock, ARDS, RRT, cardiogenic shock	> 12	Ceftriaxone and spiramycin	Piperacillin-tazobactam and gentamicin	Yes / 2
4	2018	April	Reunion	51	Male	Chronic alcoholism, tobacco use, cirrhosis	Septic shock, ARDS, RRT	> 12	Cefotaxime and spiramycin	Piperacillin-tazobactam	No
5	2019	January	Reunion	66	Male	Chronic alcoholism, tobacco use	Septic shock, ARDS, RRT, cardiogenic shock	> 24	Amoxicillin-clavulanic acid	Piperacillin-tazobactam and amikacin	Yes / 3
6	2020	March	Reunion	55	Male	Chronic alcoholism, tobacco use, COPD	Septic shock, ARDS, RRT, cardiogenic shock	> 24	Ceftriaxone and spiramycin	Piperacillin-tazobactam and amikacin	Yes / 2
7	2022	May	Reunion	58	Male	Chronic alcoholism, tobacco use, HBP	Septic shock, ARDS, cardiogenic shock	> 24	Ceftriaxone and spiramycin	Ceftazidime and amikacin	No
8	2022	October	Reunion	59	Male	Chronic alcoholism, tobacco use, COPD	Septic shock, ARDS, cardiogenic shock	> 24	Cefotaxime and spiramycin	Piperacillin	No
